# Mutational landscapes of tongue carcinoma reveal recurrent mutations in genes of therapeutic and prognostic relevance

**DOI:** 10.1186/s13073-015-0219-2

**Published:** 2015-09-23

**Authors:** Andre Luiz Vettore, Kalpana Ramnarayanan, Gregory Poore, Kevin Lim, Choon Kiat Ong, Kie Kyon Huang, Hui Sun Leong, Fui Teen Chong, Tony Kiat-Hon Lim, Weng Khong Lim, Ioana Cutcutache, John R. Mcpherson, Yuka Suzuki, Shenli Zhang, Thakshayeni Skanthakumar, Weining Wang, Daniel SW Tan, Byoung Chul Cho, Bin Tean Teh, Steve Rozen, Patrick Tan, N. Gopalakrishna Iyer

**Affiliations:** Cancer Stem Cell Biology Program, Duke-NUS Graduate Medical School, 8 College Road, Singapore, 169857 Singaore; Laboratory of Cancer Molecular Biology, Department of Biological Sciences, Federal University of São Paulo, Rua Pedro de Toledo 669, São Paulo, 04039-032 Brazil; Laboratory of Cancer Epigenome, National Cancer Centre Singapore, 11 Hospital Drive, Singapore, 169610 Singapore; Cancer Therapeutics Research Laboratory, National Cancer Centre, 11 Hospital Drive, Singapore, 169610 Singapore; Department of Pathology, Singapore General Hospital, Outram Road, Singapore, 169608 Singapore; Department of Surgical Oncology, National Cancer Centre, 11 Hospital Drive, Singapore, 169610 Singapore; Division of Medical Oncology, Yonsei Cancer Center, Yonsei Unversity College of Medicine, 250 Seongsanno, Seodaemun-gu, Seoul 120-752 South Korea; Cancer Science Institute of Singapore, National University of Singapore, 14 Medical Drive, #12-01, Singapore, 117599 Singapore; Department of Psychiatry and Behavioral Sciences, Duke University Medical Center, Durham, NC 27710 USA; Cancer Therapeutics and Stratified Oncology, Genome Institute of Singapore, 60 Biopolis Street, Genome #02-01, Singapore, 138672 Singapore

## Abstract

**Background:**

Carcinoma of the oral tongue (OTSCC) is the most common malignancy of the oral cavity, characterized by frequent recurrence and poor survival. The last three decades has witnessed a change in the OTSCC epidemiological profile, with increasing incidence in younger patients, females and never-smokers. Here, we sought to characterize the OTSCC genomic landscape and to determine factors that may delineate the genetic basis of this disease, inform prognosis and identify targets for therapeutic intervention.

**Methods:**

Seventy-eight cases were subjected to whole-exome (n = 18) and targeted deep sequencing (n = 60).

**Results:**

While the most common mutation was in *TP53*, the OTSCC genetic landscape differed from previously described cohorts of patients with head and neck tumors: OTSCCs demonstrated frequent mutations in *DST* and *RNF213*, while alterations in *CDKN2A* and *NOTCH1* were significantly less frequent. Despite a lack of previously reported *NOTCH1* mutations, integrated analysis showed enrichments of alterations affecting Notch signaling in OTSCC. Importantly, these Notch pathway alterations were prognostic on multivariate analyses. A high proportion of OTSCCs also presented with alterations in drug targetable and chromatin remodeling genes. Patients harboring mutations in actionable pathways were more likely to succumb from recurrent disease compared with those who did not, suggesting that the former should be considered for treatment with targeted compounds in future trials.

**Conclusions:**

Our study defines the Asian OTSCC mutational landscape, highlighting the key role of Notch signaling in oral tongue tumorigenesis. We also observed somatic mutations in multiple therapeutically relevant genes, which may represent candidate drug targets in this highly lethal tumor type.

**Electronic supplementary material:**

The online version of this article (doi:10.1186/s13073-015-0219-2) contains supplementary material, which is available to authorized users.

## Background

Oral squamous cell carcinoma (OSCC) accounts for 270,000 new cancer cases and 145,000 deaths annually, the majority of which occur in developing countries [1]. Of these, two-thirds are oral tongue squamous cell carcinoma (OTSCC) [2, 3]. Current treatment strategies involve a multimodality approach involving surgery, chemotherapy, and radiotherapy. Unfortunately, despite advances in detection and treatment, 5-year overall survival (OS) rates for OTSCC remain alarmingly low [4]. Currently, for all cancers arising in the head and neck [head and neck squamous cell carcinoma (HNSCC)], cancers of the tongue remain among the worst in terms of prognosis [4].

OTSCC traditionally was thought to be a cancer afflicting older males with longstanding exposure to tobacco and alcohol. In recent decades, however, there appears to have been a shift in epidemiology towards a higher proportion of never-smokers and younger patients, with an increase in incidence among women [5–9]. Although the basis of this epidemiological trend remains elusive, what is definitely worrisome is that the cancers arising in this ‘atypical population’ tend to be more aggressive and resistant to conventional treatment [6, 8, 9].

The expanding application of next-generation sequencing technology to cancer has revolutionized our ability to identify genetic alterations essential for carcinogenesis and to uncover therapeutic vulnerabilities. Coupled with the rapidly expanding array of therapeutic compounds targeting key somatic oncogenic ‘driver’ alterations, these technologies promise a new era for targeted therapy [10]. This approach has made significant headway in a number of cancers, such as breast and lung carcinoma, where alterations in the EGFR/ERBB pathways proved to be the main drivers that could be targeted with specific small molecules [11, 12].

Unfortunately, it has been difficult to translate the same success across to HNSCC. Recent large-scale genome sequencing efforts have validated *TP53* as the commonest mutation in HNSCC, but have failed to identify any single ‘oncogenic driver’ gene or family that is conventionally targetable, although some studies are examining the possibility of targeting tumor suppressors using synthetic lethal approaches [13–17]. Notably, the majority of these publications, including The Cancer Genome Atlas (TCGA), have focused on HNSCC as a single entity [18]. However, epidemiological evidence suggests that biological differences and clinical peculiarities do exist between different head and neck sites, subsites and populations affected by this disease [1, 19, 20]. For example, certain molecular alterations appear to be present in higher frequencies or unique to certain sites/subsites: these include *PIK3CA* mutations that are more prevalent in human papillomavirus (HPV)-induced oropharyngeal cancer, and alterations in *USP9X*, *MLL4*, *ARID2*, *UNC13C*, and *TRPM3* in gingiva-buccal cancers [18, 21, 22]. Even within the oral cavity alone, the distinct clinical behaviors of tumors arising from the oral tongue, buccal, alveolar, and hard palate suggests that these should be examined as different and separate entities [9]. Specifically, in the oral cavity, tumors arising from the anterior tongue are known to be more infiltrative and metastatic and hence have the poorest outcome compared with tumors in other subsites [4, 23, 24]. Furthermore, it is now emerging that the mutation spectrum of many malignancies can be strongly influenced by ethnic background, encompassing population genomic variations, environmental exposures, and lifestyle practices [25–27]. For example, in a recent International Cancer Genome Consortium (ICGC) analysis of gingiva-buccal cancers, specific genetic changes appeared to be unique to patient ethnicity (Indian) or disease etiology (betel nut chewing) [22]. Taken collectively, these findings raise the important need to consider HNSCC not as one condition but as a spectrum of different diseases, thus requiring genomic analysis of specific cohorts that are well-defined with regards to disease subsite and the at-risk patient population [28].

To date, the majority of previous genetic studies focused on OTSCC have been confined to single gene analysis or used limited gene panels, and thus have not provided a comprehensive landscape [29, 30]. A recent publication that examined the mutational spectra and copy number variations of oral tongue tumors showed that tumors from young patients and non-smokers appear to be genomically similar to those of older patients and smokers [17, 28]. These data suggest that genomic alterations are unable to account for the changing demographics in OTSCC. In this study, we set out to define the mutational landscape of OTSCC in an Asian population, with a secondary focus on identifying driver mutations with a potential for therapeutic targeting. Our objectives were to determine whether tumors arising from the tongue had a distinct genetic landscape, if these differences were population specific or correlated with any epidemiological factors (e.g., gender), and to determine the proportion of alterations with therapeutic potential.

## Methods

### Patients and bio-specimens

Patients were identified from an institutional database of a consecutive series of patients treated at the National Cancer Centre Singapore between 1998 and 2012. Included patients were confirmed to have a histological diagnosis of squamous cell carcinoma involving the anterior tongue, with complete clinico-pathologic data and fresh frozen tumor samples available, with primary surgical treatment at the National Cancer Centre Singapore. Only patients aged 21 years or above diagnosed with primary OTSCC and not previously treated were included in the study. Treatment decisions were made in weekly multi-disciplinary meetings and recorded prospectively. All patients were treated with primary surgery followed by adjuvant therapy, if applicable. Samples for 78 patients were retrieved from the Singhealth Tissue Repository and tumor content confirmed to be greater than 50 % by a board-certified pathologist (TKL). For the ‘discovery set’, germline DNA from blood was used as a reference for detecting somatic alterations. Medical records were reviewed for standard demographic data, risk factors, clinical, radiological, and histo-pathologic information, treatment parameters, and outcome of the disease. Smoking was defined as use of tobacco, chewable or smoked, for at least 1 year continuously. Patient characteristics are shown in Additional file 1.

### Ethical issues

In accordance with the Helsinki Declaration of 1975, standardized written consent for use of clinical material (which covers tumor tissue, blood, or other clinical specimens) and clinico-pathologic data for research was obtained at the time of surgical excision. Both this study and the tissue collection/consent protocols have been approved by the Singhealth Centralized Institutional Review Board (CIRB 2007/438/B).

### DNA extraction, library construction, exome capture, and sequencing

Genomic DNA was isolated from tumor specimens and blood samples using the DNeasy Blood and Tissue kit (Qiagen, Crawley, UK) according to the manufacturer’s instructions. Two micrograms of genomic DNA was sheared to 200 bp using a Covaris E Series ultrasonic solubilizer (Covaris Inc., Woburn, MA, USA) and after adapter ligation the resultant library was subjected to DNA capture using either the Agilent SureSelect Human All Exon 50 MB V3 (Agilent, Santa Clara, CA, USA) or a custom-designed capture library (SureSelect XT2 Target Enrichment System, Agilent) designed to capture the coding sequences of 465 genes. Library construction and DNA capture were carried out according to the manufacturer’s instructions. The custom 465-gene SureSelect capture probes were designed using the Agilent SureDesign package [31]. The concentration and size distribution of the libraries were determined on an Agilent Bioanalyzer DNA 1000 chip. Captured libraries were sequenced on a HiSeq2000 platform (Illumina, San Diego, CA, USA). The image analysis and base calling were done using the Illumina pipeline (v.1.6) with default settings.

### Sequence processing and variant calling

Read pairs were aligned to the National Center for Biotechnology Information (NCBI) human reference genome GRCh37 (hg19) using Burrows-Wheeler Aligner (BWA) software [32]. PCR duplicates removal was done using SAMTools [33]. After duplicate removal, the Genome Analysis Tool Kit (GATK) [34] was used for base quality score recalibration, local realignment around indels, and variant calling. Partek Genomics Suite v.6.6 (Partek Inc., St. Louis, MO, USA) was also used to identify somatic single nucleotide mutations in targeted exons.

To exclude common germline polymorphisms, the detected variants were checked against dbSNP 138 [35], 1000 Genomes [36], and ExAC [37] databases. In the discovery set, the variants detected in the matched normal controls were subtracted from variants detected in the tumors. In the ‘prevalence set’, due to the absence of matched normal controls, rare germline SNVs were discarded using an in-house Southeast Asian normal exome dataset, including 523 exomes (sgSNPG). For further analysis, we restricted our attention to candidate somatic SNVs that were in the capture target and present inside exons or at canonical splice sites, with a variant depth ≥5 and depth in the normal sample ≥10.

Variants that passed this filtering were annotated using several gene-transcript databases (Consensus CDS, RefSeq, Ensembl and UCSC [38]). Amino acid changes were determined based on the longest gene transcript. Somatic nonsynonymous single-nucleotide mutations were analyzed using PolyPhen2 [39] in order to predict their impact on the protein function. To identify significantly mutated genes, the SMG test in the genome MuSiC (Mutational Significance in Cancer) suite was used with default parameters [40]. Microindels were detected using GATK IndelGenotyperV2. For further analysis we only considered microindels that were in a target and in an exon or at a canonical splice site. Several filters were applied to reduce false positives: we retained microindels that (1) were not in a simple repeat, (2) had variant depth ≥5 with at least one read on each strand, (3) had depth in the normal sample ≥10, (4) were contained in ≥5 % of the reads, (5) had average base quality of all bases inside the microindel and ±5 bases around the microindel ≥25, (6) had an average number of mismatches in the reads containing the microindel ≤4, and (7) had an average fraction of mismatched bases in reads containing the microindel ≤0.2. Candidate somatic alterations were also assessed by visualizing sequencing data for each alteration using the Integrative Genomics Viewer (IGV; version 2.3.25) [41]. The COSMIC database was used to annotate known cancer variants. Raw sequence data have been deposited at the European Genome-phenome Archive [42], accession number [EGA:EGAS00001001329].

### Sanger sequencing validation

To validate somatic mutations by Sanger sequencing, PCR amplicons encompassing the candidate mutation sites were sequenced in cancer tissues and their normal tissue counterparts using the ABI PRISM BigDye Terminator Cycle Sequencing Ready Reaction kit (Applied Biosystems, Foster City, CA, USA) on an ABI 3730 xl DNA Analyzer (Applied Biosystems). PCR primers for amplification and sequencing were designed by targeting regions immediately flanking the predicted variant using Primer3 software [43]. The chromatograms were analyzed using the CodonCode Aligner software [44] and manual review.

### Gene selection for targeted sequencing

For the target-exome sequencing, 465 protein-coding genes were selected according to the following criteria: (a) genes identified as somatically altered in the discovery set based on occurrence in at least two samples (28 genes); (b) genes identified as somatically altered in the discovery set based on occurrence in at least one sample, but harboring single-nucleotide variations (SNVs) called by both caller algorithms, GATK and PGS (164 genes); (c) genes described as frequently altered in head and neck tumors in earlier published literature [13, 14] (45 genes); (d) genes with significant mutation frequency across 279 HNSCC tumors present in TCGA dataset (42 genes); and (e) recognized cancer genes included in the Ion AmpliSeq™ Comprehensive Cancer Panel (Life Technologies) (186 genes) (Additional file 2).

### Statistical analyses

Statistical analysis was performed using the statistical software SPSS 19.0 for Windows (IBM, New York, NY, USA). In order to assess the association between the anatomical and pathological variables of patients and the molecular findings in the tumor samples, the chi-square or Fisher’s exact tests were performed, as appropriate. Mutational frequency differences between the OTSCC and TCGA data were assessed using chi-square test, followed by the Benjamini-Hochberg procedure for multiple testing corrections. Survival curves were calculated by the Kaplan-Meier method and differences between groups were compared using the log-rank test. OS was measured as the time interval between the date of the initial treatment for the primary tumor and the date of the last follow-up or death, while disease-free survival (DFS) was defined as the time interval between the date of initial treatment and the date of the diagnosis of the first recurrence. For evaluation of the independent contribution of significant clinical and molecular variables on DFS or OS, all factors with significance in Kaplan-Meier analysis (*P* ≤ 0.2) were tested in the multivariate analyses using the Cox proportional hazard model. Results were calculated with 95 % confidence intervals (CIs). For all analysis we considered statistical significance when *P* value < 0.05. In the pathway analysis, the *P* values were calculated by Gene Set Enrichment Analysis (GSEA) algorithm by estimating the hypergeometric distribution of overlapping genes over all genes in the gene universe.

### URLs

UCSC Genome Bioinformatics [38], CodonCode Aligner software [44], dbSNP [45], 1000 Genomes Project [46], Exome Variant Server [47], COSMIC [48], ExAC database [37], cBioPortal for Cancer Genomics [49], MSigDB pathways database [50], Genomics of Drug Sensitivity in Cancer [51], Clinical trials database [52], Drugs approved by US Food and Drug Administration (FDA) [53], European Genome-phenome Archive [42].

## Results

### Patient characteristics

Eighteen OTSCC patient tumor and matched blood samples were used for the discovery set, and 60 tumor samples were used in the prevalence set (clinico-pathologic data for both cohorts is shown in Additional file 1). The prevalence set was used for all subsequent clinical correlations. In the latter cohort, approximately half (51.7 %; n = 31) were smokers, 63.3 % (n = 38) were males, with a median age of 55.5 years (range 21–89 years). All patients in the prevalence cohort underwent surgery as the primary modality of treatment, and 26 (43.3 %) received adjuvant radiation or chemo-radiation therapy. Approximately half presented with locally advanced disease (pT3/T4, 48.3 %, n = 29) and had pathologic evidence of nodal metastasis (pN+, 46.7 %, n = 28) at presentation. Patients were followed up for a median of 36 months (range 1–190 months). During this time period, there were 17 (28.3 %) recurrences and 13 (21.7 %) deaths.

### Overview of OTSCC somatic mutation profile

To identify somatic mutations, we performed whole-exome capture and massively parallel DNA sequencing on paired DNA samples (tumor and matched white blood cells) from 18 OTSCCs (discovery set). From each sample, we sequenced around 16 billion bases, resulting in an average 93-fold coverage of each base in the targeted regions (range 70–115), with 94 % of DNA sequences being covered sufficiently (≥10×) for confident variant calling (Additional file 3).

Overall, 1,092 nonsynonymous somatic variations affecting 750 genes were detected, of which 992 were somatic SNVs and the remaining were somatic insertions or deletions (indels) (Additional file 4). The co-mutation plot for the discovery set is provided in Additional file 5. Of the single-nucleotide substitutions, 888 (81.3 %) were predicted to be missense mutations, 68 (6.2 %) nonsense mutations, and 36 (3.4 %) splice site mutations. The number of variants (SNVs and indels) per patient ranged from 11 to 105, with a mean of 61 mutations per sample, similar to previous oral cavity whole-exome sequencing studies (ranging from 29 to 85) [22, 28]. The alterations were verified manually using IGV. Subsequent technical validation by Sanger sequencing confirmed the presence of mutations in 28 genes (28/29, true-positive rate of 96.6 %), demonstrating that the algorithms used (GATK and PGS) for identifying mutations were reliable. Overall, the whole-exome sequencing of the discovery set indicated 28 genes harboring somatic coding mutations in two or more oral tongue tumors (a list of these 28 genes is presented in Additional file 6).

Nucleotide sequence contexts of somatic mutations can be indicative of specific mutagenesis mechanisms occurring in tumor cells [54–56]. Analyzing synonymous and non-synonymous alterations, we observed elevated rates of C > T transitions, particularly at CpG positions, consistent with deamination of 5-methyl-cytosine to uracil by APOBEC or other endogenous processes. C > G mutations were also common (Additional file 7). We visually examined the somatic mutation spectra of the 18 exome-sequenced tumors and performed principal component analysis to search for possible systematic differences in spectra according to several clinical factors: age, smoking status, gender, HPV status, ethnicity, and recurrence. These analyses did not suggest any systematic differences according to any of these categories (Additional file 6; see “Discussion”).

Copy number variant analyses were performed as previously described [57]. The results are presented in Additional files 8, 9, and 10. We identified 30 amplified regions and 56 regions with recurrent deletions in the discovery set. Focal amplifications in chromosome 7 harboring the *MET* oncogene were seen in 7 of 18 samples, while deletions were commonly seen on chromosome 3 (9/18 samples), involving *VHL, SETD2, PBRM1, MLH1*, and *BAP1*. All regions of gains and losses, with their corresponding tumor-associated genes are indicated in Additional files 8 and 9. There were no correlations between copy number variants and smoking status, age, gender, ethnicity, or recurrence.

### Evaluating the frequency and distribution of somatic mutations by target sequencing

To further investigate the precise frequency and distribution of somatic mutations in an expanded OTSCC cohort, we designed a panel with 465 genes including (a) the 28 candidate genes frequently mutated in the discovery set, (b) genes previously described as frequently mutated in HNSCC [13, 14], and (c) recognized cancer genes that were manually curated (a list of all genes included in the target panel is presented in Additional file 10). These genes were sequenced in a prevalence set of 60 treatment-naive snap-frozen OTSCC specimens by targeted sequencing with an average depth of 136× (Additional file 11).

For the prevalence set, we used a consecutive patient series treated in a standardized manner in order to make meaningful conclusions on epidemiology and outcome, and only tumor samples were available for this cohort [58]. Therefore, an alternative strategy to discriminate germline SNVs in the absence of normal tissue DNA was adopted, following previously described studies [59, 60]. We created a high-fidelity tumor-only analysis pipeline that relied on two steps. First, we excluded variants found in either public databases of germline SNVs [dbSNP (build 132), 1000 Genomes, and EVS (6500-SI-V2)] [35, 36, 47] or in an in-house database of germline SNVs drawn from 523 normal Asian exomes. We initially also planned to exclude variants described in the beta version of the ExAC database, but preliminary tests showed that this filtering steps removes a significant proportion of the *TP53* alterations in our cohorts (23 % discovery set and 22 % prevalence set). Furthermore, the examination of the TCGA data for HNSCC also revealed that 22 % of the *TP53* mutations described there were also present in the ExAC database, with a significant proportion of damaging missense mutations. Given these findings, we did not utilize the ExAC database in the final analysis. Variants present in the COSMIC database (v.52) were kept regardless of their match in the other databases, because dbSNP in particular contains some known oncogenic somatic mutations. Second, we excluded variants with allele frequencies >0.41. The rationale for this criterion is that somatic mutations usually present with variant read proportions substantially less than 0.5 in next-generation sequencing data, due to admixture of genomes from non-malignant cells in a tumor sample. By contrast, germline variants have read proportions of either 1 (for homozygous variants) or ~0.5 (for heterozygous variants) in regions where the tumor genome is diploid. We note that copy number gains or losses in tumor genomes may alter the expected variant read proportions of germline variants, so this two-step approach will not always exclude germline variants. Conversely, genomic copy number gains may sometimes cause somatic mutation to have read proportions greater than 0.5, so this two-step approach sometimes incorrectly excludes somatic mutations. As discussed below, we estimated the accuracy of this approach in a set of HNSCCs with matched normal DNA.

To validate this two-step tumor-only filtering protocol and to verify the extent to which it would retain rare germline alterations amidst bona fide somatic mutations, we applied it to both the discovery set and an independent cohort of 16 HNSCC exomes for which normal matched controls were available. For the discovery set, this algorithm showed a high degree of reliability and accuracy with a positive predictive value (PPV) of 92 % and negative predictive value (NPV) of 96 % (with 89 % sensitivity and 97 % specificity). Further validation of this tumor-only protocol in an independent cohort of 16 non-subsite specific HNSCC samples also showed good reproducibility with PPV of 94 % and NPV of 89 % (with 73 % sensitivity and 98 % specificity). These control analyses support the notion that our tumor-only filtering protocol does not lead to excessive numbers of germline variants miscalled as somatic mutations.

**Fig. 1:**
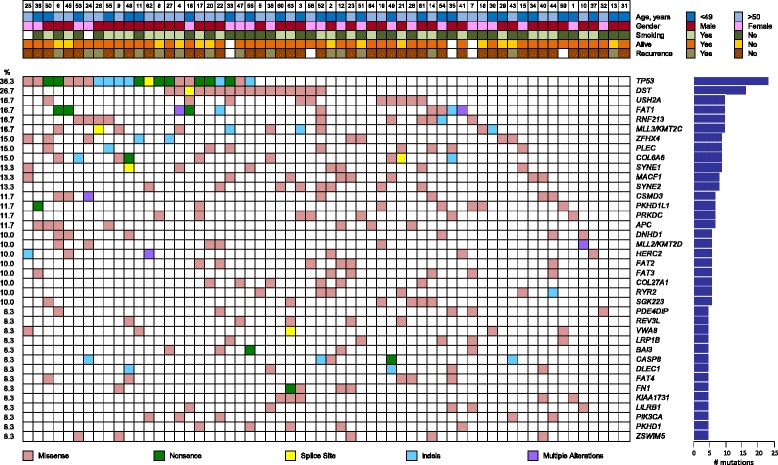
Mutation plot summary of 60 oral tongue squamous cell carcinoma patients showing frequently mutated genes. The top plot shows the key clinical parameters, below which the mutation status of the recurrently mutated genes for each tumor is indicated. Somatic mutations are colored according to functional class according to the legend below the plot. Prevalence is indicated as number of mutations in the graph on the right and mutational frequency is given on the left of the mutation plot

After applying the tumor-only filtering protocol to the prevalence set, 380 genes were detected as harboring SNVs or indels. All mutations identified in the prevalence set are shown in Additional file 12. Unsurprisingly, the most prevalent genetic alteration detected in this cohort comprised a broad spectrum of *TP53* mutations (38.3 %). Other genes recurrently mutated include *DST* (26.7 %), *RNF213* (16.7 %), *USH2A* (16.7 %), *FAT1* (16.7 %), *MLL3*/*KMT2C* (16.7 %), *COL6A6* (15.0 %), *ZFHX4* (15.0 %), *PLEC* (15.0 %), and *SYNE1* (13.3 %) (Fig. 1). We examined expression levels of these ten genes using HNSCC RNA-seq data from TCGA and our own previously published microarray data [61], both of which confirm that the genes listed here are known to be expressed in OTSCC (data not shown).

**Fig. 2:**
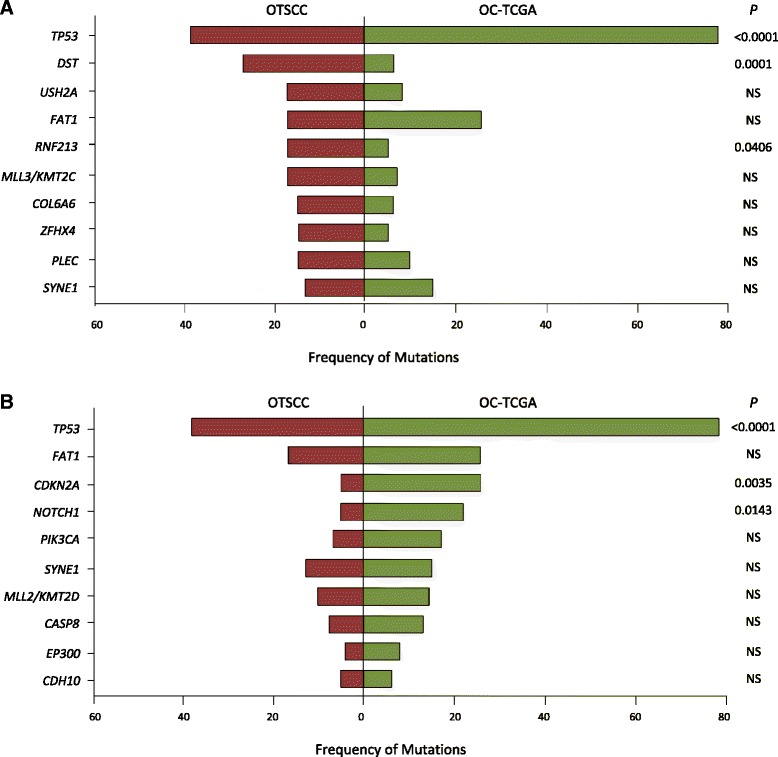
The frequency of commonly mutated genes in the current cohort of oral tongue squamous cell carcinoma (OTSCC) compared with oral cavity squamous cell carcinoma from TCGA (OC-TCGA). **a** The most frequently mutated genes in the OTSCC cohort. **b** The most frequently mutated genes in the OC-TCGA cohort. Significance is determined by multiple hypothesis testing using chi-square test, followed by the Benjamini-Hochberg procedure. *NS* not significant

This pattern of Asian OTSCC genetic mutations was compared with the profile of 172 exomes from oral cavity tumors available from TCGA (OC-TCGA) [48]. One major caveat to this comparison is that there are fundamental differences between the two cohorts examined here in terms of tumor site (tongue versus oral cavity), disease stage, ethnicity (Asian versus North American) and environmental exposure. Single gene comparisons showed that mutation frequencies of the following genes were higher in Asian OTSCCs compared with OC-TCGA: *DST* (*P* < 0.001), *RNF213* (*P* = 0.012), *COL6A6* (*P* = 0.049) and *ZFHX4* (*P* = 0.031). Even after multiple hypothesis correction, mutation frequencies remained higher in Asian OTSCCs for *DST* (*P* = 0.001) and *RNF213* (*P* = 0.033) (Fig. 2). In contrast, OC-TCGAs exhibited more frequent mutations in *TP53* (*P* < 0.001), *CDKN2A* (*P* = 0.005), and *NOTCH1* (*P* = 0.019). Critically, no significant differences were observed in the mutation rates of many other genes frequently mutated in both cohorts (*CASP8*, *CDH10*, *EP300*, *FAT1*, *MLL2*/*KMT2D*, *MLL3*/*KMT2C*, *NSD1*, *PIK3CA*, *PLEC*, *SYNE1*, and *USH2A*). These similarities in mutation frequency in the latter genes provide a sound validation of and positive control for our methodology, for both the experimental approach (tumor content, library creation, and sequencing) and bioinformatics pipeline.

### Analysis of significantly altered pathways

**Fig. 3:**
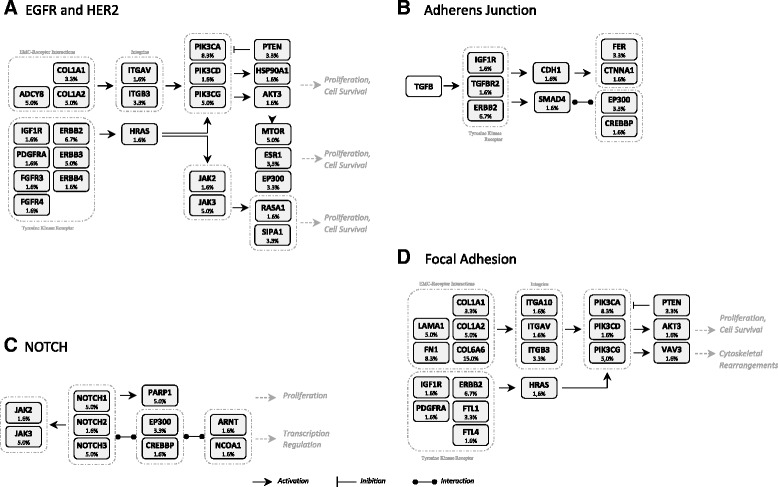
Pathway map showing molecular circuits implicated in OTSCC. **a** EGFR and HER2. **b** Adherens junction. **c** NOTCH. **d** Focal adhesion. Numbers in each box represent the percentage of tumors harboring mutations in the respective gene

Pathway analysis was performed to examine the association between mutation profiles and core canonical pathways in Asian OTSCC, using either the MSigDB pathways database [62] or compared with manually curated cancer-related pathways [63, 64]. Putative cancer-related pathways achieving statistical significance, as determined by GSEA, are listed in Additional file 13. Among the most prominently mutated pathways were the epidermal growth factor receptor (EGFR), ERRB, mitogen-activated protein kinase (MAPK), and NOTCH signaling pathways, as well as adherens junction and focal adhesion pathways (Fig. 3; Additional file 14). Overall, 55 % of the OTSCC samples had mutations in at least one gene affecting the MAPK pathway, while EGFR, Notch, and ERBB signaling families where modified in 52 %, 32 % and 23 % of cases, respectively. Genes involved in cell junction organization and focal adhesion pathways were mutated in 22 % and 53 % of OTSCC cases, respectively. Other core canonical signaling pathways frequently found mutated in OTSCC included mammalian target of rapamycin (mTOR), transforming growth factor β–SMAD, chromatin remodeling genes, KIT, β-catenin and apoptosis, many of which may have therapeutic implications. Correlations between significantly altered pathways and clinical characteristic/outcomes are presented in Additional file 15.

### Notch pathway mutations

**Fig. 4:**
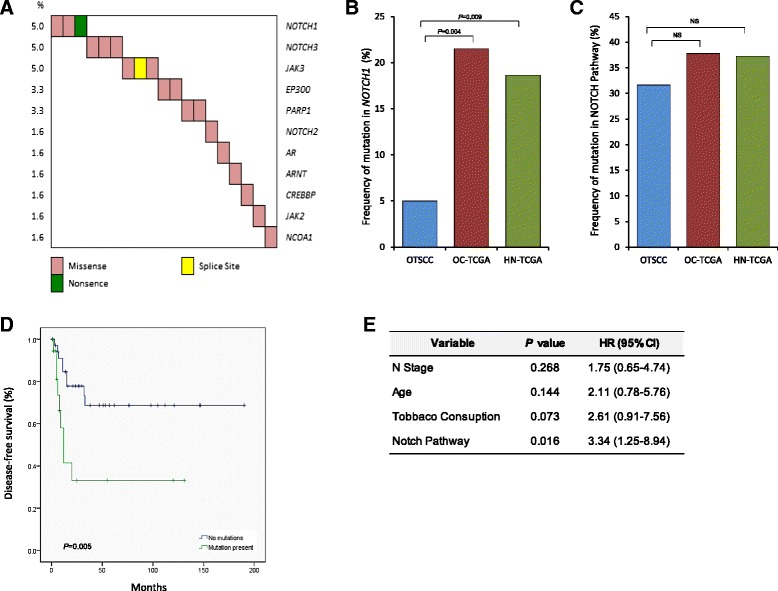
**a** Co-mutation map showing alterations in genes involved in the Notch pathway in 19 (32 %) oral tongue squamous cell carcinoma (OTSCC) samples. Gene names and mutation rates are as indicated on either side of the plot, while the type of mutation follows the legend below. **b**, **c** Comparison between OTSCC, oral cavity cohort from TCGA (*OC-TCGA*) and entire head and neck cohort of TCGA (*HN-TCGA*) for *NOTCH1* mutation frequency and alterations in the entire Notch pathway (11 genes), respectively. *P* value based on t-test as indicated if significant or *NS* if not significant. **d** Kaplan-Meier plot showing DFS estimates according to the mutational status of the Notch pathway. *P* value is based on log-rank test. **e** Results of multivariate analysis of selected prognostic factors for DFS in OTSCC by Cox proportional hazards model. *HR* hazard ratio

As stated above, we found the Notch pathway to be altered in a high proportion of the Asian OTSCCs. However, classical single-gene *NOTCH1* mutations, previously reported in other studies, were notably absent from our cohort (only three cases have *NOTCH1* mutations). Nevertheless, pathway analyses demonstrated that the Notch signaling pathway was altered in OTSCC in the same proportion as previously reported for HNSCC [14] or detected in oral cavity tumors and HNSCC by TCGA (Fig. 4). Genes recurrently altered in this pathway include *AR*, *ARNT*, *EP300*, *CREBBP*, *JAK2*, *JAK3 NCOA1*, *NOTCH2*, *NOTCH3*, and *PARP1.* In total, the Notch signaling pathway was altered in 32 % (19/60) of the OTSCC cases, with all somatic events following a mutually exclusive pattern (Fig. 4).

Interestingly, mutations in the Notch signaling pathway were enriched in women compared with men (*P* = 0.04; Additional file 15). More importantly, a striking difference in DFS was observed between OTSCC cases with mutations in the Notch pathway compared with those without, where the former had significantly poorer outcomes (*P* = 0.005; Fig. 4; Additional file 13). Based on multivariate models that included clinico-pathologic correlates associated with prognosis (age, tobacco consumption, N stage and extra-capsular spread), modifications in the Notch pathway remained an independent predictor for DFS. Patients with Notch pathway mutations were 3.3 times more likely to die with recurrent disease compared with those who did not have these alterations [*p* = 0.016, hazard ratio (HR) = 3.3, 95 % CI = 1.24–8.94; Fig. 4]. Analysis of TCGA data for oral cavity suggests a similar trend towards poorer OS in patients with alterations in this pathway, however this was not statistically significant (*p* = 0.13; data not shown).

Gene expression microarray data were available for a limited number of these samples: Notch pathway altered (n = 9) versus Notch pathway wild type (n = 21) [61]. GSEA analysis of gene expression confirmed that the presence or absence of these somatic events resulted in significant alteration of two Notch associated pathways (Nguyen Notch1 Targets DN, *p* = 0.004; PID Notch pathway, *p* = 0.011), confirming that these alterations truly modulate Notch signaling (Additional file 16). These orthogonal data suggest that the Notch pathway is modulated as predicted in the cases with mutations in Notch pathway.

### Chromatin remodeling gene family

**Fig. 5:**
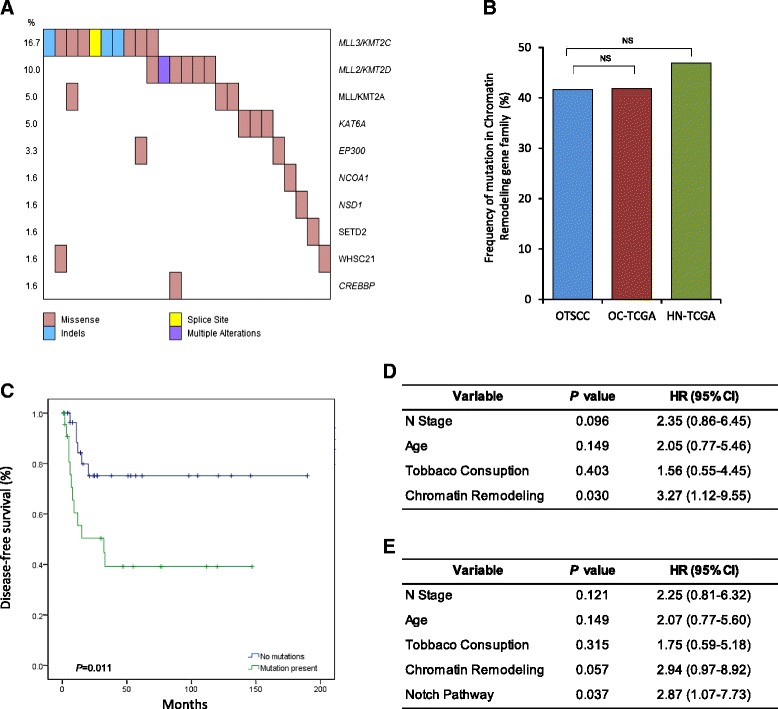
**a** Co-mutation map showing alterations in genes involved in chromatin remodeling in 25 (42 %) oral tongue squamous cell carcinoma (OTSCC) samples. Gene names and mutation rates are as indicated on either side of the plot, while the type of mutation follows the legend below. **b** Comparison between OTSCC, the oral cavity cohort from TCGA (*OC-TCGA*) and the entire head and neck cohort of TCGA (*HN-TCGA*) for frequency of genetic alterations in ten chromatin remodeling genes. *P* value based on t-test; *NS* not significant. **c** Kaplan-Meier plot showing DFS estimates according to the mutational status of the chromatin remodeling family. *P* value is based on log-rank test. **d**, **e** Results of multivariate analysis of selected prognostic factors for DFS in OTSCC by Cox proportional hazards model. *HR* hazard ratio

There has been significant interest in the role of chromatin remodeling enzymes in a range of cancers, and examination of this gene set revealed alterations in 42 % of the OTSCC cohort, similar to data from TCGA for the oral cavity (42 %) and for all HNSCC (47 %) (Fig. 5). Chromatin remodeling genes frequently altered in OTSCC included *CREBBP*, *EP300*, *KAT6A*, *MLL*/*KMT2A*, *MLL2*/*KMT2D*, *MLL3*/*KMT2C*, *NCOA1*, *NSD1*, *SETD2*, and *WHSC1.* Moreover, most of the mutations in this gene family were mutually exclusive, especially mutations affecting the MLL family of histone methyltransferases (Fig. 5). Mutations affecting chromatin remodeling genes were significantly more frequent in older patients (*p* = 0.03; Additional file 15) and patients with mutations have significantly poorer DFS compared with those who did not (*p* = 0.01; Fig. 5). Based on multivariate models, modifications in this gene set remained an independent predictor for DFS. Patients with mutations in this pathway were 3.3 times more likely to die with recurrent disease compared with those who did not have these alterations (*p* = 0.03, HR = 3.3, 95 % CI = 1.12–9.55; Fig. 5). Again, analysis of TCGA data for tongue cancers suggests a similar trend towards poorer OS in patients with alterations in this pathway, although this was not statistically significant (*P* = 0.11; data not shown).

Remarkably, multivariate analysis including both the Notch pathway and chromatin remodeling genes as variables showed that alterations in the Notch pathway remained as an independent predictor of recurrence. Patients harboring mutations in at least one member of this signaling pathway were 2.9 times more likely to develop recurrence and die (*P* = 0.037, HR = 2.9, 95 % CI = 1.07–7.73; Fig. 5). Furthermore, this same multivariate model also revealed a tendency towards to significance for the presence of mutations in chromatin remodeling genes (*P* = 0.057, HR = 2.9, 95 % CI = 0.97–8.91; Fig. 5).

### Analysis of significantly altered actionable mutations

**Fig. 6:**
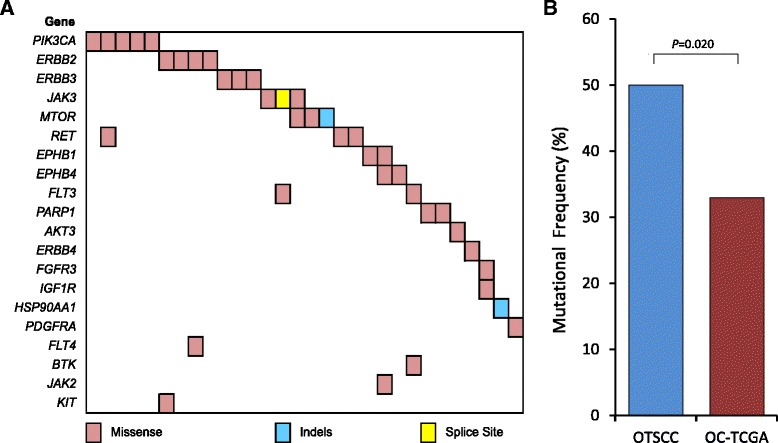
**a** Co-mutation map showing alterations in drug targetable genes in 30 (50 %) oral tongue squamous cell carcinoma (OTSCC) samples with gene names indicated on the left. **b** Comparison between OTSCC and oral cavity cohort from TCGA (*OC-TCGA*) for frequency of genetic alterations in 20 drug targetable genes. *P* value based on t-test

Finally, the mutation data were also used to characterize alterations in drug targetable genes. In this analysis, manually curated gene sets were assembled based on genes known to have similar functions/pathways, gene families, or overlapping cross-sensitivity to available targeted compounds [53, 65, 66]. This group of 20 genes included receptor tyrosine kinases, ERBB, JAK-STAT, MAPK-AKT, and phosphoinositide 3-kinase (PI3K) signaling, for which therapeutic agents are currently in clinical use or available in the trial setting. Based on this list, 50 % of OTSCC samples harbored modifications in at least one of these genes, and the majority were mutually exclusive (Fig. 6). The majority of the PIK3CA mutations (four out of five) are known to be activating mutations, while about half the mutations in the ERBB family of genes (three out of six) occur in regions that affect signaling (extracellular domain or tyrosine kinase domain) [30, 67]. Based on Polyphen2 analysis, a significant proportion of the other mutations in this set of ‘druggable’ genes are predicted to be damaging to the respective protein function. Of note, the frequency of these ‘druggable mutations’ is significantly higher in OTSCC compared with that detected in the oral cavity tumor exomes analyzed by TCGA (*P* = 0.020; Fig. 6).

## Discussion

To date, most cancer genome studies on OSCC have regarded this entity as a homogenous disease. However, differences in biological behavior already hint that OSCC is not a uniform condition [4, 9, 20], likely requiring dissection by populations affected, anatomic subsite and disease etiology [22, 25, 27, 30]. This population/subsite/etiology-based approach requires rigorous sample collection and exclusion of a significant number of cases that do not fit the selection criteria. One of the first papers that used this approach was the Indian ICGC group, who used exome analysis for gingiva-buccal oral squamous cell carcinoma (OSCC-GB). In this study, apart from detecting mutations in genes previously associated with HNSCC (*TP53*, *NOTCH1*, *FAT1*, and *CASP8*), they also identified recurring alterations in genes specific to this disease (*USP9X*, *MLL4*/*KMT2B*, *ARID2*, *UNC13C*, and *TRPM3*) [22].

In a similar fashion, our study focused on the mutational landscape of squamous cell cancers arising from the single subsite of oral (or anterior) tongue in a South-East Asian population (OTSCC). More importantly, as this cohort of patients was treated in a uniform manner at a single tertiary institution, data from this study can also be used to draw meaningful conclusions based on clinical correlates. Our patient cohort exhibits several features that are representative of the evolution of this disease in Asia. First, there is an almost equal proportion of smokers and non-smokers. Second, the male:female ratio is approaching equivalence. Third, the median age of disease has been declining over the past three decades and is now around 55 years. Reassuringly, mutational analysis of our OTSCC cohort identified frequent nucleotide alterations in *SYNE1*, *FAT1*, *MLL2*/*KMT2A*, *MLL3*/*KMT2C*, *PIK3CA*, and *CASP8* at similar frequencies reported by TCGA and ICGC. However, there were significantly fewer mutations in *TP53*, *NOTCH1*, and *CDKN2A.* Mutations in the *RAS* genes were extremely infrequent (*HRAS*, n = 1/60, with no mutations in *KRAS* and *NRAS*) and mutations in *EGFR* notably absent. In contrast, several less well-characterized genes (*DST* and *RNF213*) were mutated at higher rates in Asian OTSCC than previously reported. Most of the unique genes detected here were also not seen in the ICGC study, which focused on an Indian population with tobacco- and betel nut-associated OSCC-GB, reinforcing the need to carry out similar studies in different populations. Indeed, we hypothesize that the unique genetic alterations in both studies can be attributable to the etiology of disease, differences in the (Asian) population examined or the distinct biological features of the disease subsites (in this case oral tongue). As predicted, these genetic alterations also differed from HPV-driven cancers involving the base of tongue, which had a higher frequency of activating mutations in *PIK3CA*, with loss of *TRAF3* and amplification of *E2F1* [18]. In a similar vein, patterns attributable to etiology and specific populations have been observed for other cancer types [27].

The reason for this changing epidemiology in a disease (OTSCC) classically seen in older, male smokers remains unknown, and one of the major objectives of this study was to determine the genetic basis for this ‘emerging phenotype’. Our analysis of OTSCC found no specific mutations unique to younger patients or non-smokers, although there was a tendency towards more frequent mutations in *MLL2*/*KMT2A* and *PIK3CA* in older patients. Moreover, the mutational signature in our cohort did not reveal a tobacco-induced signature in OTSCC cases grouped by age (young versus old) or by smoking status, which is similar to findings by Pickering et al. [28]. The latter recently reported whole-exome sequencing analysis of two independent cohorts of tongue tumors and found that mutational spectrum were similar between young and old patients. Further, they also observed that the types of base changes observed in the young cohort were similar to those in the old cohort even though they differed in smoking history, and no smoking signature could also be found in OTSCC. These findings, now supported by two independent studies, suggests that tobacco smoke, despite being an established risk factor for OTSCC, may act primarily as a tumor promoter while having only a minor impact on the initiation of tongue cancers [68].

Analysis of the OTSCC data at the gene set level provided valuable insights into pathways that potentially drive OTSCC carcinogenesis. We were initially surprised that *NOTCH1* mutations were relatively infrequent in this cohort, although one previous study in esophageal squamous cell cancer had shown that *NOTCH1* mutations are uncommon in Asian patients compared with matched Caucasian populations [69]. However, pathway analysis confirmed that the Notch pathway was affected in a third of our OTSCC cases by mutations that were mutually exclusive, distributed across 11 distinct genes along the pathway. These alterations were more common in women, and of concern, they conferred a poorer prognosis in these patients regardless of other factors or mutations, even on multivariate analysis. The role of Notch as a tumor suppressor pathway in OSCC has been suggested by several studies [13, 14, 70]. Activated *NOTCH1* has an anti-proliferative effect in tongue tumor cells through down-regulation of Wnt/β-catenin signaling, inducing apoptosis and cell cycle arrest [71]. Furthermore, in a tongue cancer model using mouse xenografts, the presence of *NOTCH1* caused a dramatic reduction in tumor size compared with tumors in which this gene was absent [17]. This apparent discordance between infrequent *NOTCH1* mutations per se and a still defective Notch pathway through other gene mutations in 32 % of OTSCC patients highlights the importance of deregulating the Notch-driven epithelial cell differentiation program in OTSCC through a range of convergent targets.

Analyses of the chromatin remodeling gene family led to similar conclusions. Interestingly, we observed a significantly higher frequency of *MLL3*/*KMT2C* mutations (16 %) in our cohort compared with mutations in the *MLL* homologs *MLL2*/*KMT2A* in OC-TCGA (Western/predominantly Caucasian population) [21] and in *MLL4*/*KMT2D* in OSCC-GB [22]. Damaging mutations in *MLL2*/*KMT2A* were more frequently identified in smokers in our cohort, while the mutations in *MLL4*/*KMT2D* seem to be unique to the Indian population studied by the ICGC [22]. The higher frequency of *MLL3*/*KMT2C* mutations in our cohort may therefore be a phenomenon associated with never-smoker OTSCC populations, and represents another example of convergent evolution on a critical pathway in OTSCC carcinogenesis.

Despite advances in technology, clinical outcomes have not changed significantly over the past three decades for OSCC. However, analysis of our cohort showed that at least half of the patients harbored potentially targetable mutations in signal transduction pathways, where targeted compounds are already available. These results have several important implications. Firstly, the mutation frequencies of these genes in our population were significantly higher than in previously reported studies, and this pattern should therefore be taken into consideration in future trial designs. Second, previous pessimism regarding the low occurrence of sensitizing mutations in HNSCC should not be translated to any cohort until similar studies have been conducted in this population/subsite/etiology-based approach. Interestingly, there was a similar conclusion by the ICGC group regarding OSCC-GB [22], who also similarly identified higher rates of potentially actionable mutations.

Beyond OTSCC, our study is also notable from a technical standpoint. To date, most cancer sequencing studies have utilized tumor and normal matched pairs to distinguish de novo somatic mutations from existing germline variants. In this study, we needed to obtain tumor representation from a consecutive series of patients presenting with tongue cancer, treated in a standardized manner with clinic-pathologic and outcome data available, with no bias in patient selection (prevalence set), to enable robust clinico-pathological analysis. In this process, however, it was not possible to obtain matched normal blood from every patient in this prevalence set. This is a very common scenario encountered in the translational research domain, motivating the need to develop approaches to identify somatic mutations in the absence of matched normal controls. In our study, we addressed this challenge by using, in addition to commonly used pipelines (e.g., 1000 Genomes, dbSNP, EVS), a locally assembled 523-patient normal sample dataset to discriminate the germline SNVs. One major concern was that this approach may not remove all rare germline alterations, erroneously classifying these as somatic variations. To test the validity of this approach, we determined the PPV, NPV, specificity, and sensitivity of the filtering algorithm by applying the tumor-only filtering protocol on the discovery set as well as an independent cohort of HNSCC cases (comprising 16 tumor–normal matched pairs). The data for the discovery set show that the methodology was extremely reliable, with PPV and NPV over 90 %. Even after correcting for these accuracy data, the differences observed between our cohort and TCGA remains significant. Further validation of this approach can be inferred from the fact that we found similar frequencies of mutations in a number of genes previously detected by TCGA, including *USH2A*, *FAT1*, *PLEC*, *SYNE1*, *PIK3CA*, *MLL2*/*KMT2A*, *MLL3*/*KMT2C*, *CASP8 COL6A6*, and *ZFHX4*. There are a few other studies in the literature where ‘tumor-only’ protocols have been used. In a study conducted at the Sanger Centre, investigators were able to identify base substitutions and small insertions/deletions in the exome data from 738 patients with myelodysplastic syndrome by comparison against one unmatched normal sample [59]. However, no data were presented on the accuracy of this approach. Along the same line, a Japanese study identified mutations in a cohort of 97 Japanese lung adenocarcinoma patients by filtering out germline variants present in an external exome dataset composite by 217 normal control exomes from individuals with the same ethnic background [60]. The latter study reported accuracy rates of 64 %, establishing the validity of this approach. It is likely that the accuracy rates for any tumor-only pipeline will improve with increasing numbers of control/normal sample data from which germline alterations can be subtracted, and more importantly represent the same population distribution in which the primary analysis is carried out. The latter approach likely accounts for the accuracy of our approach, which focused on the same patient population. It is likely that with increasing numbers of normal samples deposited into ‘open source’ databases like ExAC, these algorithms can be further refined. Furthermore, the availability of these databases and algorithms allow future studies to proceed with retrospective sample collections, regardless of matched normal samples, at half the cost for sequencing. This approach also obviates the issue of dealing with patient germline data and the inherent ethical dilemma that this generates.

## Conclusions

The present study identified genomic alterations enriched in pathways that are specific to OTSCC. Of note, mutations in genes in the Notch pathway and the chromatin remodeling family were associated with poor prognosis in individuals with oral tongue tumors. OTSCC is a frequently lethal cancer with few effective therapeutic options. Despite the genomic alterations previously observed in different HNSCCs, no new therapeutic targets have been identified, and, therefore, surgery, radiation, and cisplatin still remain as standard therapy for the disease. To our knowledge, this is the first study identifying targetable alterations in a high proportion of OTSCCs. This finding is important with respect to the development of novel approaches to patient treatment, which could be used in combination with standard therapy.

## Additional files

Additional file 1: Table S1. Clinico-pathologic characteristics of discovery and prevalence cohorts. (DOC 59 kb) Additional file 2: Table S7. List of 465 genes evaluated in the prevalence set (target-exome sequencing). (XLSX 25 kb) Additional file 3: Table S2. Summary of whole exome sequencing results for 18 tongue tumor samples and normal-matched controls in the discovery set. (XLS 15 kb) Additional file 4: Table S3. Somatic mutations detected in the discovery set using whole-exome sequencing). (XLS 228 kb) Additional file 5: Figure S1. Mutation plot summary of 18 oral tongue squamous cell carcinoma patients examined in the ‘discovery set’. The top plot shows the key clinical parameters, below which the mutation status of the recurrently mutated genes for each tumor is indicated. Somatic mutations are colored according to functional class and color coded according to the legend below the plot. Prevalence is indicated as number of mutations in the graph on the right and mutational frequency is given in the left of the mutation plot. (PPT 423 kb) Additional file 6: Table S4. List of selected genes harboring somatic coding mutations in two or more OTSCC samples evaluated in the discovery set. (XLSX 11 kb) Additional file 7: Figure S2.
**a** Mutational signatures for each sample in the discovery set. **b** Mutational signatures found in the discovery set grouped according to clinical characteristics (age, recurrence, gender, ethnicity, and smoking status). Signatures are displayed according to the 96 substitution classification defined by the substitution class and sequence context immediately 3′ and 5′ of the mutated base. The probability bars for the six types of substitutions are displayed in different colors. The mutation types are on the horizontal axes, whereas vertical axes depict the proportions of mutations attributed to a specific mutation type. All mutational signatures are displayed on the basis of the trinucleotide frequency of the human genome. (DOC 913 kb) Additional file 8: Figure S3. Copy number variation plot summary of 18 oral tongue squamous cell carcinoma patients in the discovery set. The samples are sorted according to smokers and never-smokers as indicated. Regions of gains and losses are color coded as per the legend. (PPT 662 kb) Additional file 9: Table S5. Recurrently amplified regions in the discovery cohort. (XLSX 13 kb) Additional file 10: Table S6. Recurrently deleted regions in the discovery cohort. (XLSX 12 kb) Additional file 11: Table S8. Summary of target exome sequencing results for 60 tongue tumor samples in the prevalence set. (XLSX 14 kb) Additional file 12: Table S9. Somatic mutations detected in the prevalence set (target-exome re-sequencing). (XLS 186 kb) Additional file 13: Table S10. List of significantly mutated pathways identified in OTSCC by GSEA pathway analysis or comparisons with well-recognized cancer-related pathways. (XLS 62 kb) Additional file 14: Figure S4. Top ten significantly mutated pathways in oral tongue squamous cell carcinoma (OTSCC), as determined by examination of the Molecular Signatures Database (MSigDB) by Gene Set Enrichment Analysis (GSEA) software and comparisons with well-recognized cancer-related pathways. (PPT 137 kb) Additional file 15: Table S11. Correlations between demographic data of OTSCC patients and altered pathways. (XLS 35 kb) Additional file 16: Table S12. List of top ten significantly altered cancer-related pathways according to gene expression data comparing tumors with and without mutations in the Notch pathway (microarray data analyzed by GSEA algorithm). (XLSX 10 kb)

## Abbreviations

CI: confidence interval
DFS: disease-free survival
EGFR: epidermal growth factor receptor
GATK: Genome Analysis Tool Kit
GB: gingiva-buccal
GSEA: Gene Set Enrichment Analysis
HNSCC: head and neck squamous cell carcinoma
HPV: human papillomavirus
HR: hazard ratio
ICGC: International Cancer Genome Consortium
IGV: Integrative Genomics Viewer
indel: insertion or deletion
MAPK: mitogen-activated protein kinase
NPV: negative predictive value
OC: oral cavity
OS: overall survival
OSCC: oral squamous cell carcinoma
OTSCC: oral tongue squamous cell carcinoma
PCR: polymerase chain reaction
PPV: positive predictive value
SNV: single-nucleotide variation
TCGA: The Cancer Genome Atlas
