# Supplementary material for: Mutational landscapes of tongue carcinoma reveal recurrent mutations in genes of therapeutic and prognostic relevance
Source: Genome Med. 2015 Sep 23;7(1):98. doi: 10.1186/s13073-015-0219-2 (PMC4580363; doi:10.1186/s13073-015-0219-2)
Supplement: Additional file 1: Table S1. — Clinico-pathologic characteristics of discovery and prevalence cohorts. (DOC 59 kb) [file 13073_2015_219_MOESM1_ESM.doc]

**Additional Table 1** - Clinico-pathologic characteristics of discovery and prevalence cohorts.

|  | **Discovery Set (n=18) % (n)** | **Prevalence Set**  **(n=18) % (n)** |
| --- | --- | --- |
|  |  |  |
| **Age, years** |  |  |
| <49 | 61.1 (11) | 46.7 (28) |
| >50 | 38.9 (7) | 53.3 (32) |
| Median | 51.6 | 55.5 |
| Range | 22 - 87 | 21 - 89 |
|  |  |  |
| **Sex** |  |  |
| Female | 38.9 (7) | 36.7 (22) |
| Male | 61.1 (11) | 63.3 (38) |
|  |  |  |
| **Race** |  |  |
| Chinese | 72.2 (13) | 61.7 (37) |
| Indian | 16.7 (3) | 16.7 (10) |
| Malay | 11.1 (2) | 5.0 (3) |
| Others | 0.0 (0) | 16.7 (10) |
|  |  |  |
| **Smoking Status** |  |  |
| Yes | 38.9 (7) | 45.0 (27) |
| No | 44.4 (8) | 51.7 (31) |
| Unknown | 16.7 (3) | 3.3 (2) |
|  |  |  |
| **T Stage** |  |  |
| T1-2 | 33.3 (6) | 41.7 (25) |
| T3-4 | 50.0 (9) | 48.3 (29) |
| Unknown | 16.7 (3) | 10.0 (6) |
|  |  |  |
| **Lymph Nodes** |  |  |
| Negative | 44.4 (8) | 45.0 (27) |
| Positive | 55.5 (10) | 46.7 (28) |
| Unknown | 0.0 (0) | 8.3 (5) |
|  |  |  |
| **Adjuvant Radio or Chemotherapy** |  |  |
| Yes | 0.0 (0) | 43.3 (26) |
| No | 44.4 (8) | 30.0 (18) |
| Unknown | 55.6 (10) | 26.7 (16) |
|  |  |  |
| **Recurrence** |  |  |
| Yes | 50.0 (9) | 28.3 (17) |
| No | 50.0 (9) | 63.3 (38) |
| Unknown | 0.0 (0) | 8.3 (5) |
|  |  |  |
| **Survival** |  |  |
| Alive | 66.7 (12) | 73.3 (44) |
| Dead | 33.3 (6) | 21.7 (13) |
| Unknown | 0.0 (0) | 5 (3) |
|  |  |  |
