# Supplementary material for: Mutational landscapes of tongue carcinoma reveal recurrent mutations in genes of therapeutic and prognostic relevance
Source: Genome Med. 2015 Sep 23;7(1):98. doi: 10.1186/s13073-015-0219-2 (PMC4580363; doi:10.1186/s13073-015-0219-2)
Supplement: Additional file 5: Figure S1. — Mutation plot summary of 18 oral tongue squamous cell carcinoma patients examined in the ‘discovery set’. The top plot shows the key clinical parameters, below which the mutation status of the recurrently mutated genes for each tumor is indicated. Somatic mutations are colored according to functional class and color coded according to the legend below the plot. Prevalence is indicated as number of mutations in the graph on the right and mutational frequency is given in the left of the mutation plot. (PPT 423 kb) [file 13073_2015_219_MOESM5_ESM.ppt]

## Slide 1
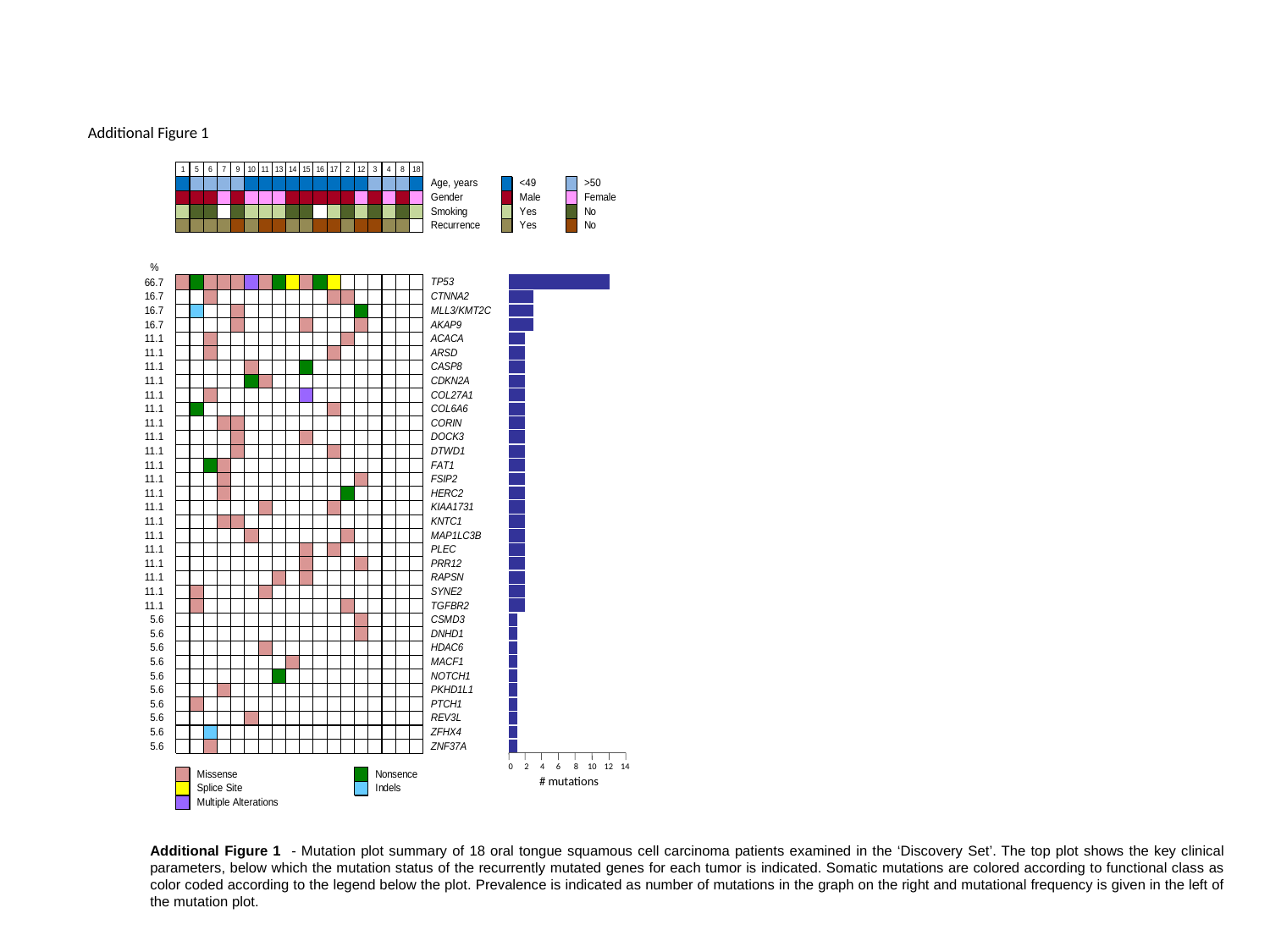

Additional Figure 1
0 2 4 6 8 10 12 14
# mutations
Additional Figure 1 - Mutation plot summary of 18 oral tongue squamous cell carcinoma patients examined in the ‘Discovery Set’. The top plot shows the key clinical parameters, below which the mutation status of the recurrently mutated genes for each tumor is indicated. Somatic mutations are colored according to functional class as color coded according to the legend below the plot. Prevalence is indicated as number of mutations in the graph on the right and mutational frequency is given in the left of the mutation plot.
