# Supplementary material for: Mutational landscapes of tongue carcinoma reveal recurrent mutations in genes of therapeutic and prognostic relevance
Source: Genome Med. 2015 Sep 23;7(1):98. doi: 10.1186/s13073-015-0219-2 (PMC4580363; doi:10.1186/s13073-015-0219-2)
Supplement: Additional file 7: Figure S2. — a Mutational signatures for each sample in the discovery set. b Mutational signatures found in the discovery set grouped according to clinical characteristics (age, recurrence, gender, ethnicity, and smoking status). Signatures are displayed according to the 96 substitution classification defined by the substitution class and sequence context immediately 3′ and 5′ of the mutated base. The probability bars for the six types of substitutions are displayed in different colors. The mutation types are on the horizontal axes, whereas vertical axes depict the proportions of mutations attributed to a specific mutation type. All mutational signatures are displayed on the basis of the trinucleotide frequency of the human genome. (DOC 913 kb) [file 13073_2015_219_MOESM7_ESM.doc]

**Additional Figure 2**

A


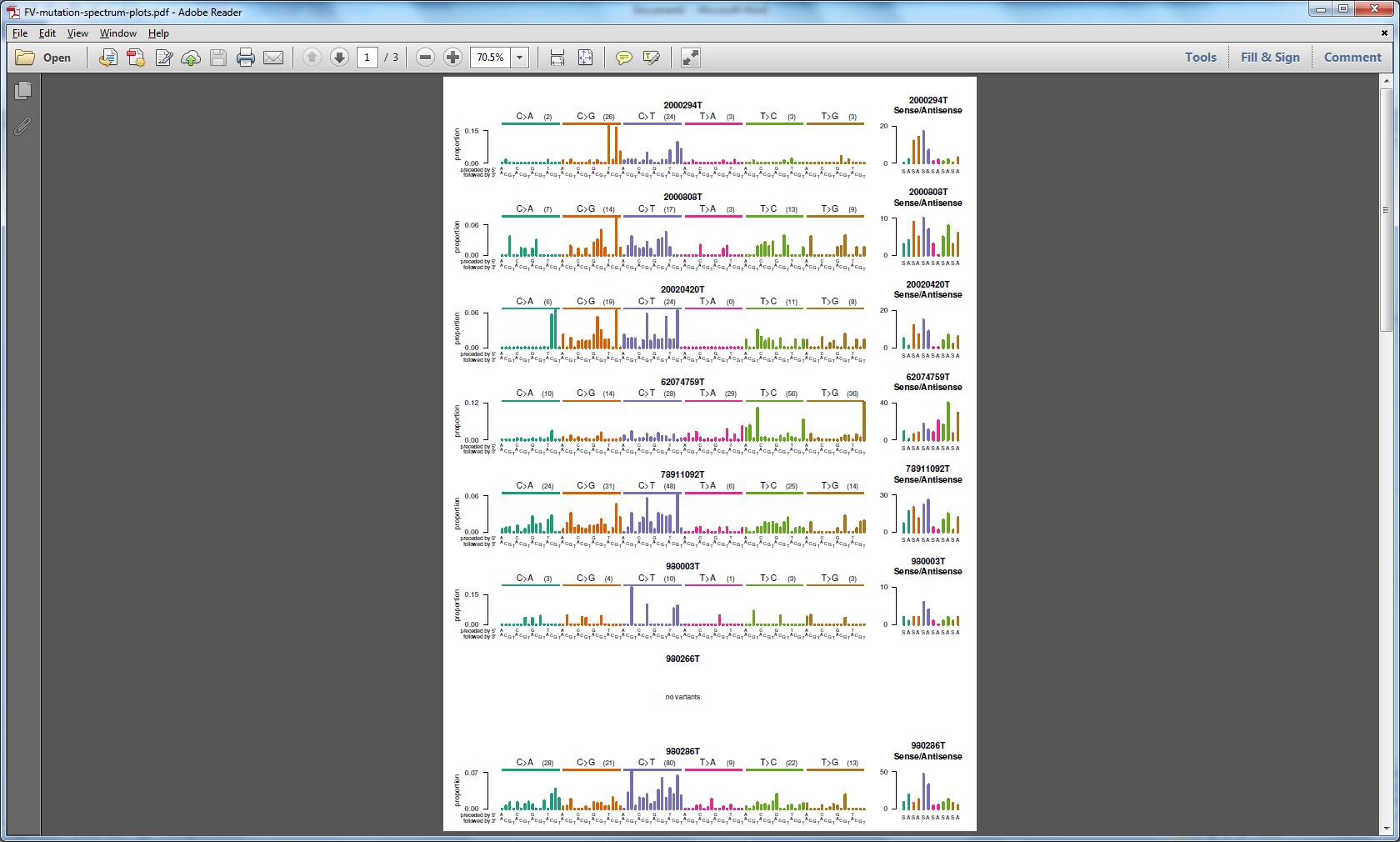


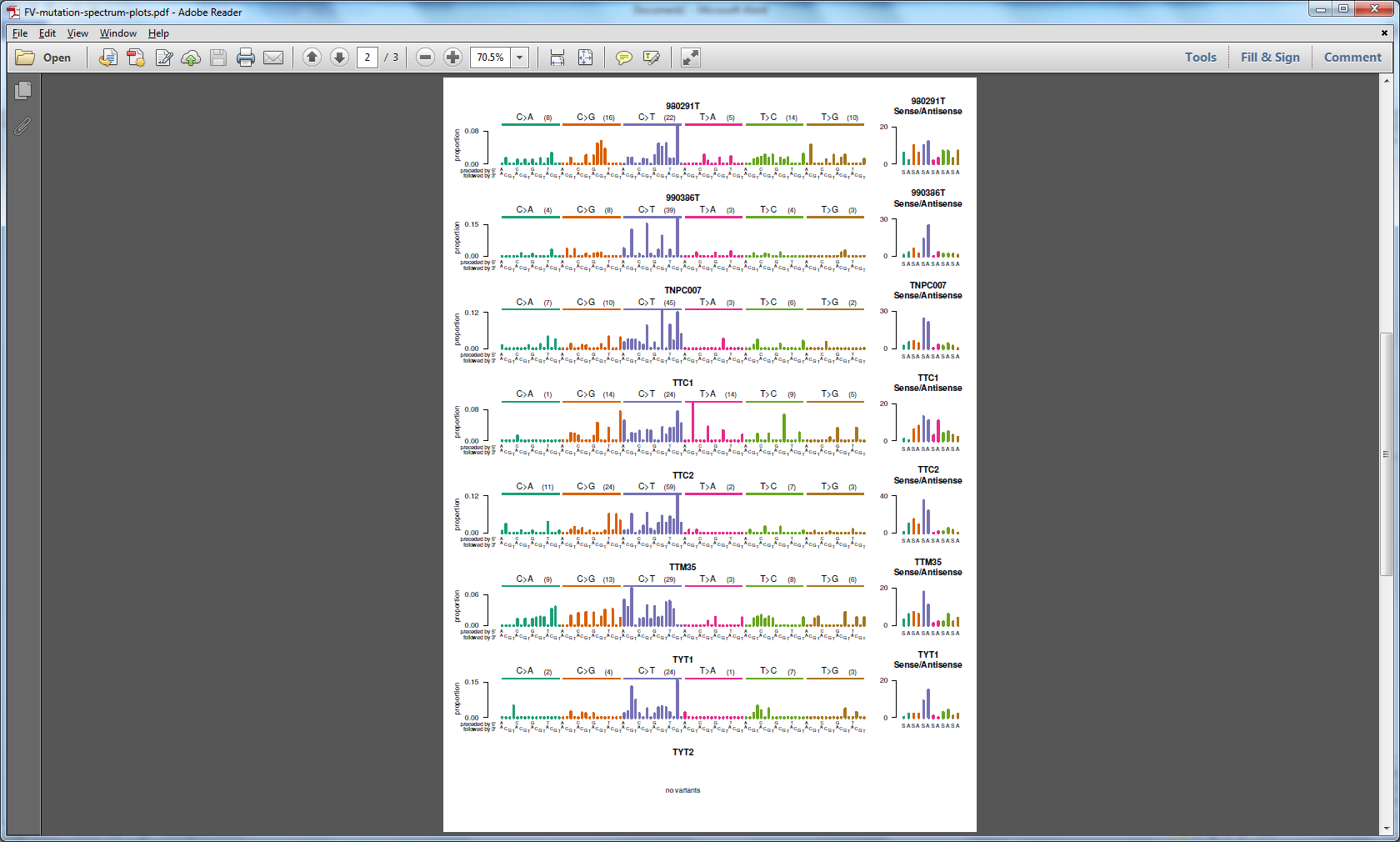


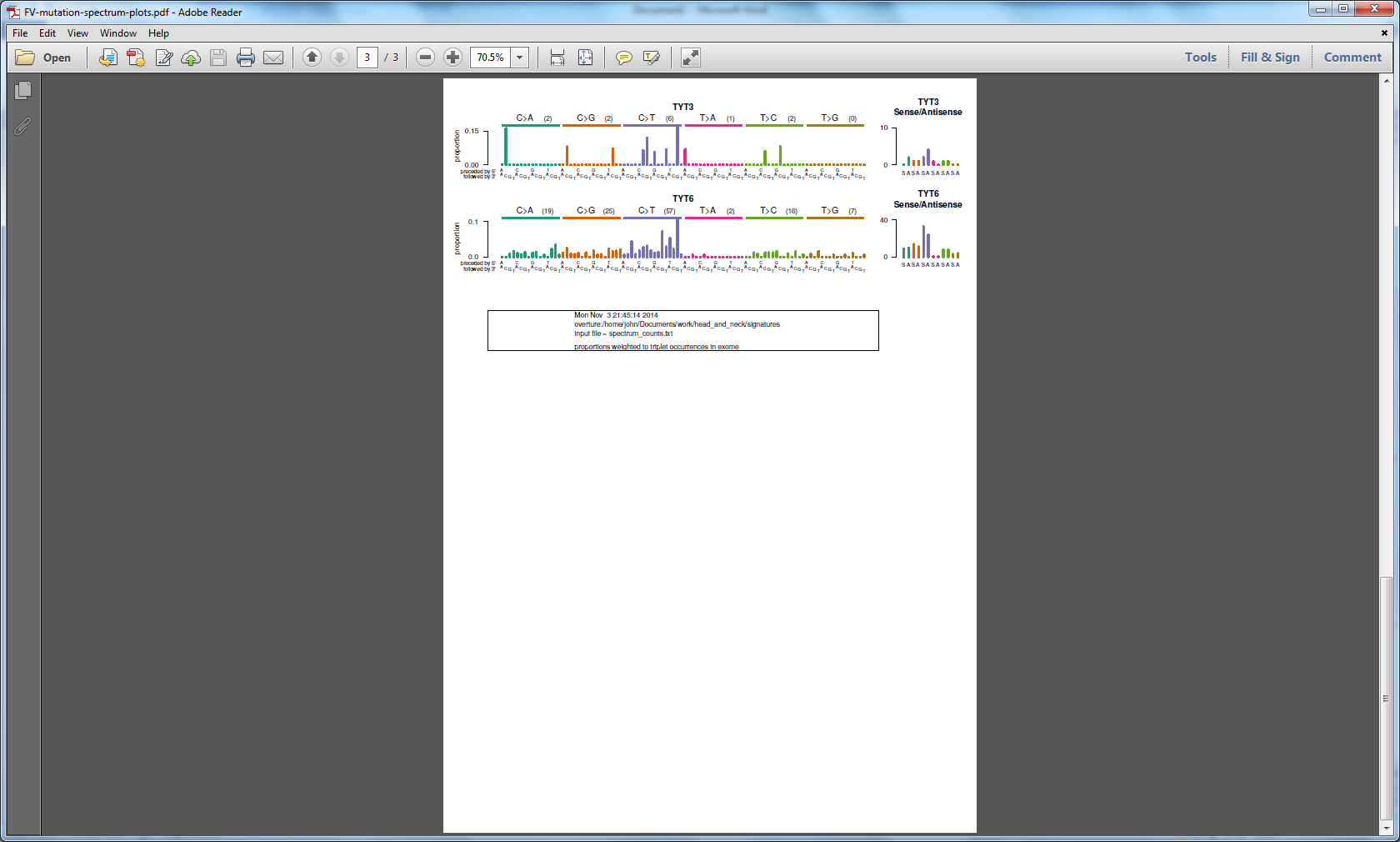


**B**


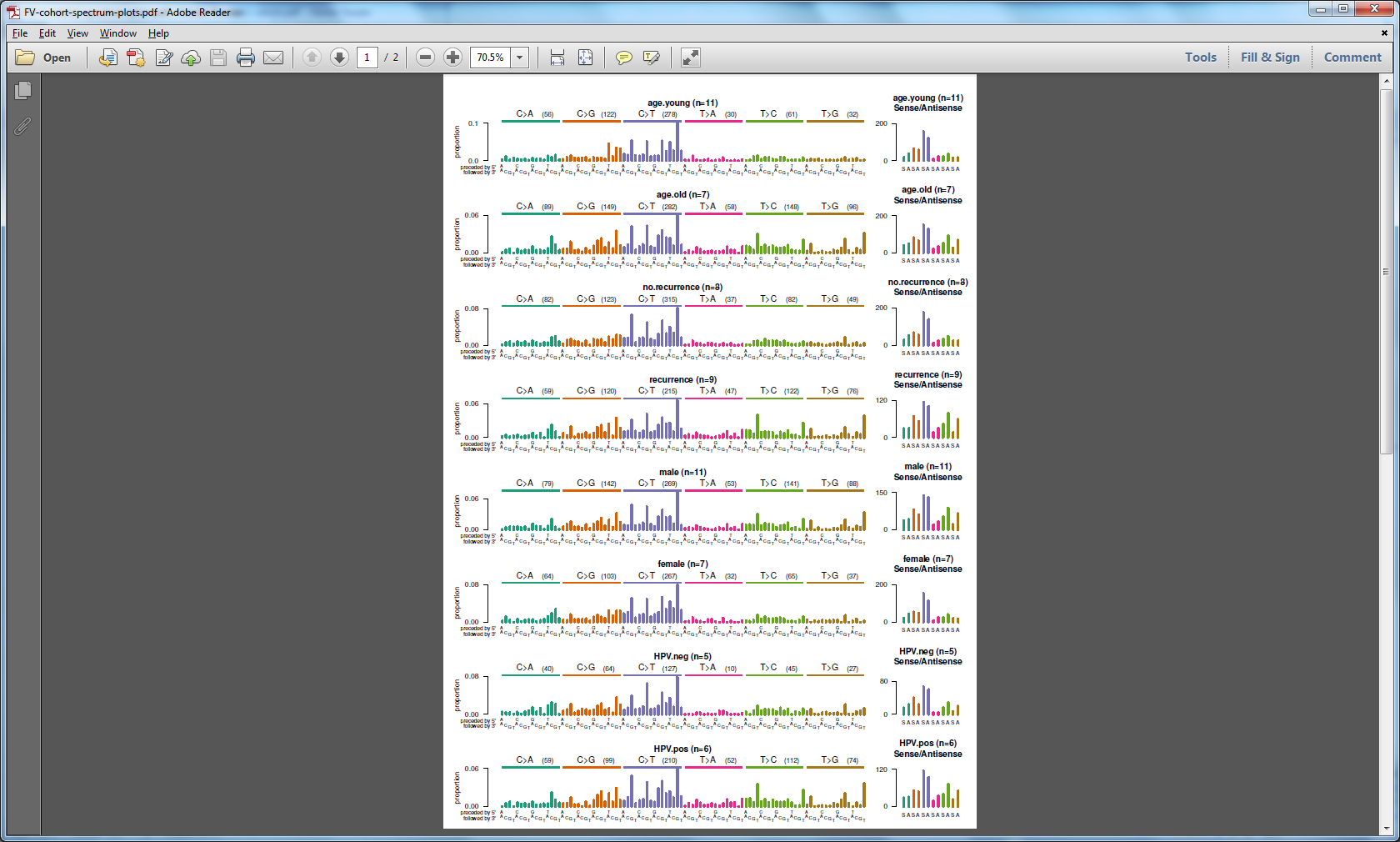


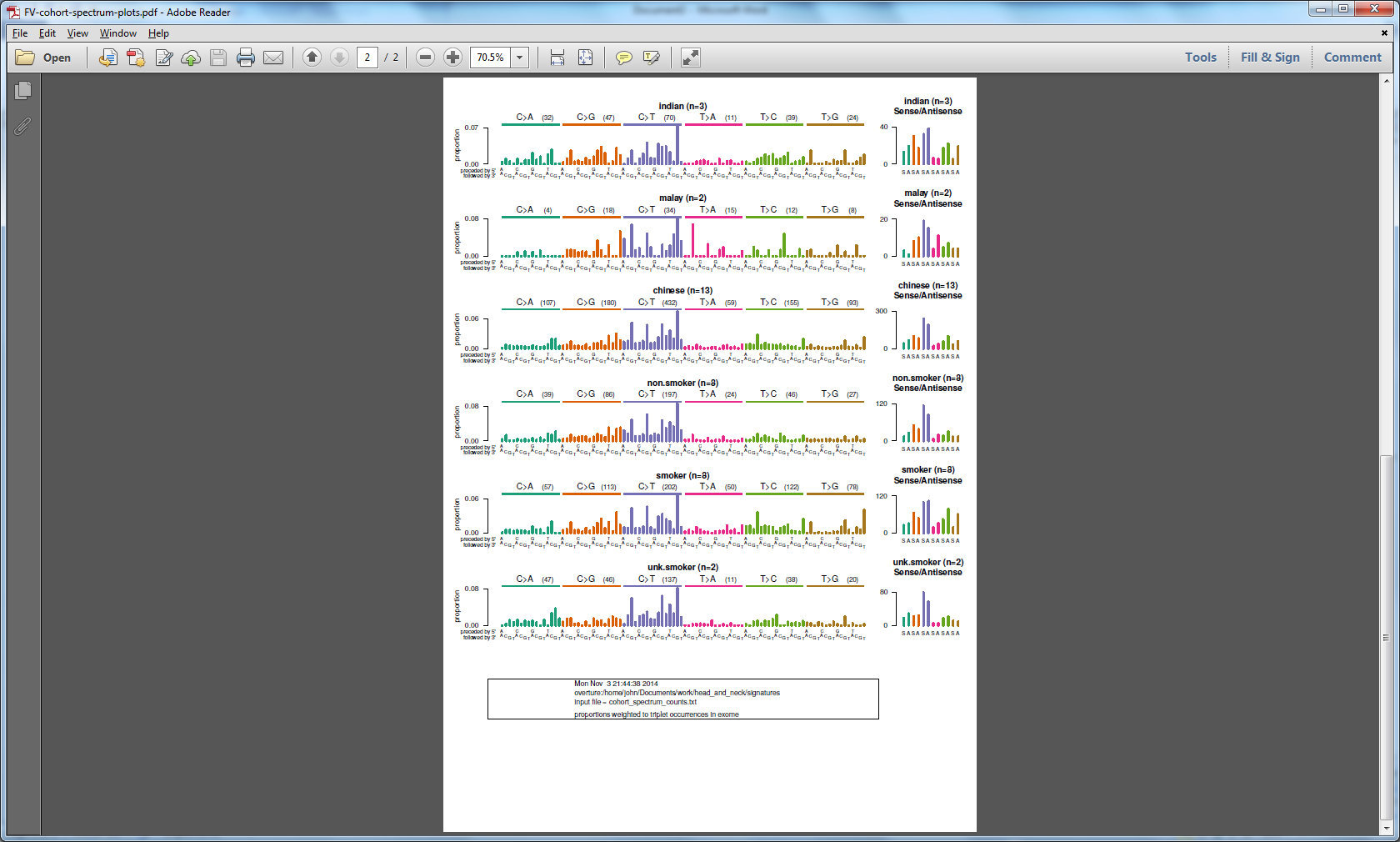


**Additional Figure 2 –** A. Mutational signatures for each sample in the discovery set. B. Mutational signatures found in the discovery set grouped according to clinical characteristics (age, recurrence, gender, ethnicity and smoking status). Signatures are displayed according to the 96 substitution classification defined by the substitution class and sequence context immediately 3’ and 5’ to the mutated base. The probability bars for the six types of substitutions are displayed in different colors. The mutation types are on the horizontal axes, whereas vertical axes depict the proportions of mutations attributed to a specific mutation type. All mutational signatures are displayed on the basis of the trinucleotide frequency of the human genome.
