# Supplementary material for: Mutational landscapes of tongue carcinoma reveal recurrent mutations in genes of therapeutic and prognostic relevance
Source: Genome Med. 2015 Sep 23;7(1):98. doi: 10.1186/s13073-015-0219-2 (PMC4580363; doi:10.1186/s13073-015-0219-2)
Supplement: Additional file 8: Figure S3. — Copy number variation plot summary of 18 oral tongue squamous cell carcinoma patients in the discovery set. The samples are sorted according to smokers and never-smokers as indicated. Regions of gains and losses are color coded as per the legend. (PPT 662 kb) [file 13073_2015_219_MOESM8_ESM.ppt]

## Slide 1
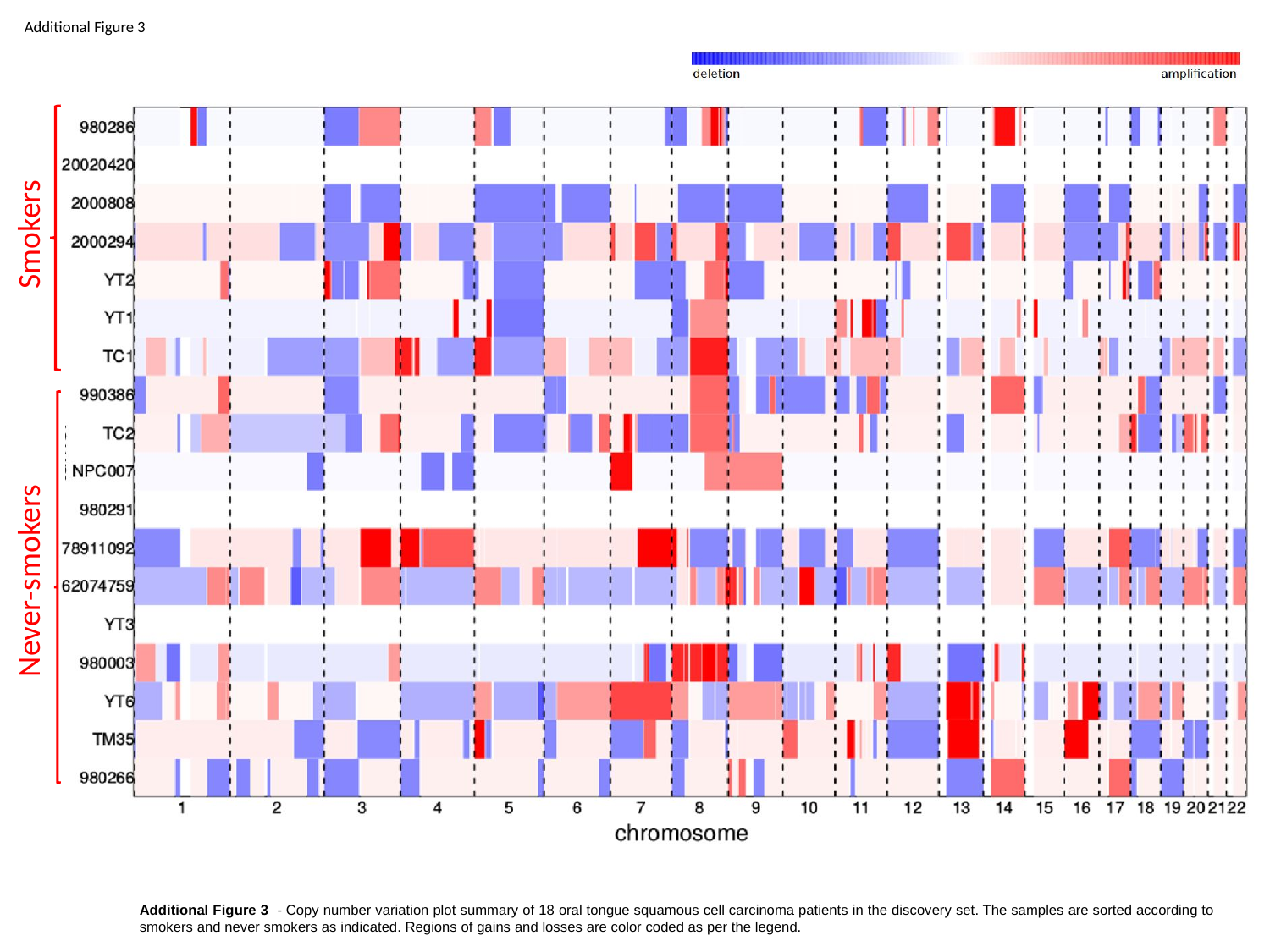

Additional Figure 3
Smokers
Never-smokers
Additional Figure 3 - Copy number variation plot summary of 18 oral tongue squamous cell carcinoma patients in the discovery set. The samples are sorted according to smokers and never smokers as indicated. Regions of gains and losses are color coded as per the legend.
