# Supplementary material for: Mutational landscapes of tongue carcinoma reveal recurrent mutations in genes of therapeutic and prognostic relevance
Source: Genome Med. 2015 Sep 23;7(1):98. doi: 10.1186/s13073-015-0219-2 (PMC4580363; doi:10.1186/s13073-015-0219-2)
Supplement: Additional file 14: Figure S4. — Top ten significantly mutated pathways in oral tongue squamous cell carcinoma (OTSCC), as determined by examination of the Molecular Signatures Database (MSigDB) by Gene Set Enrichment Analysis (GSEA) software and comparisons with well-recognized cancer-related pathways. (PPT 137 kb) [file 13073_2015_219_MOESM14_ESM.ppt]

## Slide 1
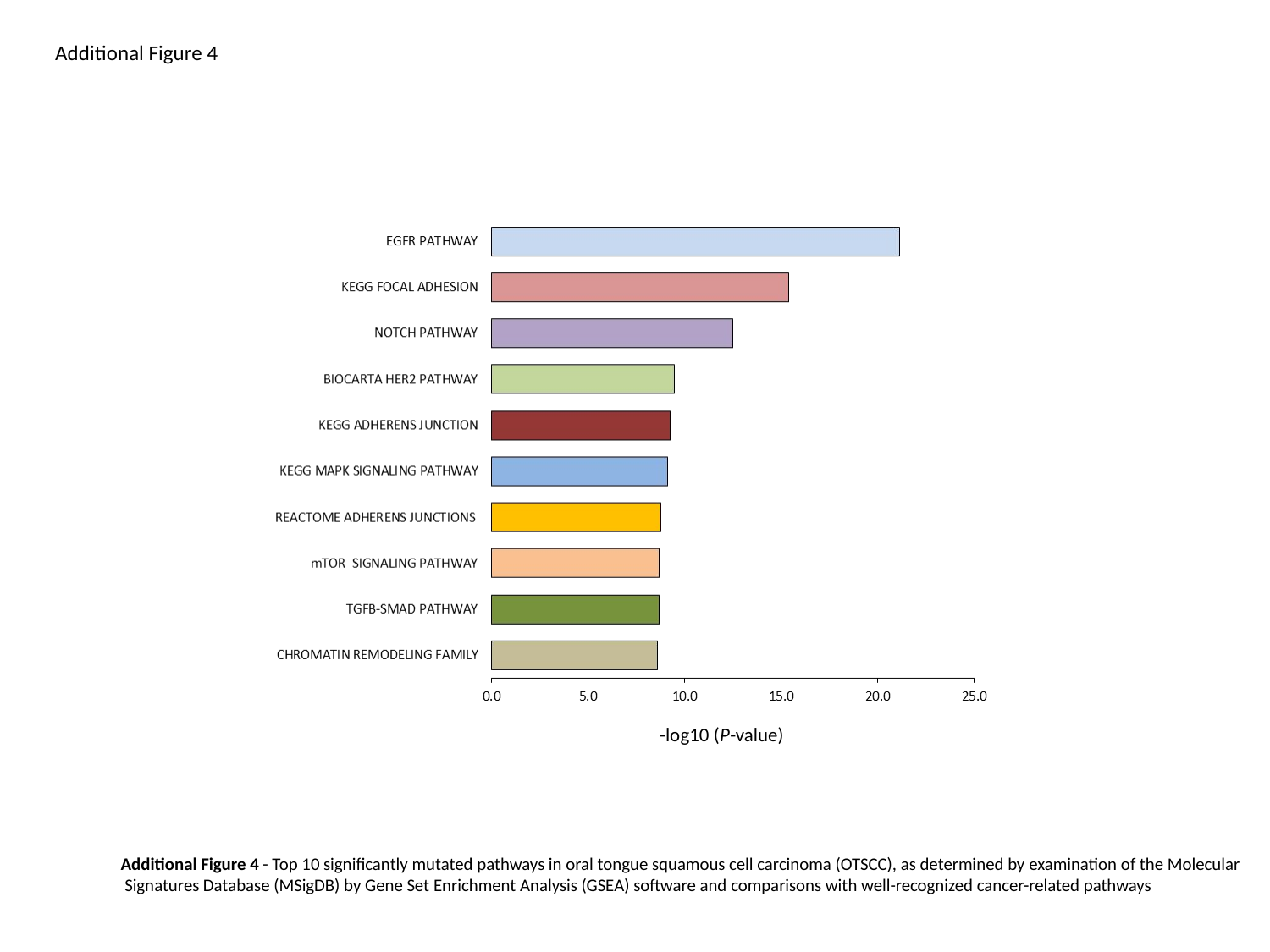

Additional Figure 4
-log10 (P-value)
Additional Figure 4 - Top 10 significantly mutated pathways in oral tongue squamous cell carcinoma (OTSCC), as determined by examination of the Molecular
 Signatures Database (MSigDB) by Gene Set Enrichment Analysis (GSEA) software and comparisons with well-recognized cancer-related pathways
